# Chinese ICU physicians’ knowledge of antibiotic pharmacokinetics/pharmacodynamics (PK/PD): a cross-sectional survey

**DOI:** 10.1186/s12909-022-03234-9

**Published:** 2022-03-14

**Authors:** Wenchao Mao, Difan Lu, Jia Zhou, Junhai Zhen, Jing Yan, Li Li, Zhejiang Provincial Critical Care Clinical Research Group

**Affiliations:** 1 Department of Critical Care Medicine, Zhejiang Hospital, Hangzhou, 310013 China; 2 The First Affiliated Hospital of Zhejiang University, Hangzhou, 310003 Zhejiang China

**Keywords:** Sepsis, Antibiotics, Pharmacokinetics, Pharmacodynamics, Intensive care units, Angoff method

## Abstract

**Background:**

Patients with sepsis have a high mortality rate, accumulated evidences suggest that an optimal antibiotic administration strategy based on pharmacokinetics/pharmacodynamics (PK/PD) can improve the prognosis of septic patients. Therefore, we assessed Chinese intensive care unit (ICU) physicians’ knowledge about PK/PD.

**Methods:**

In December 2019, we designed a questionnaire focused on Chinese ICU physicians’ knowledge about PK/PD and collected the questionnaires after 3 months. The questionnaire was distributed via e-mail and WeChat, and was distributed to ICU doctors in 31 administrative regions of China except Hong Kong, Macao and Taiwan. The passing score was corrected by the Angoff method, and the ICU physicians’ knowledge about PK/PD was analysed accordingly.

**Results:**

We received a total of 1,309 questionnaires and retained 1,240 valid questionnaires. The passing score was 90.8, and the overall pass rate was 56.94%. The pass rate for tertiary and secondary hospitals was 59.07% and 37.19%, respectively. ICU physicians with less than 5 years of work experience and resident physician accounted for the highest pass rate, while those with between 5 to 10 years of work experience and attending accounted for the lowest pass rate. The majority of participants in the Chinese Critical Care Certified Course (5C) were from Jiangsu and Henan provinces, and they had the highest average scores (125.8 and 126.5, respectively). For Beijing and Shanghai, the average score was only 79.4 and 90.9, respectively.

**Conclusions:**

Chinese ICU physicians’ knowledge about PK/PD is unsatisfactory. Therefore, it is essential to strengthen ICU physicians’ knowledge about PK/PD.

**Supplementary Information:**

The online version contains supplementary material available at 10.1186/s12909-022-03234-9.

## Background

Sepsis is one of the main causes of death in the intensive care unit (ICU) [1, 2]. According to statistics published in 2017, the incidence of sepsis is 48.9 million cases per year, and its mortality rate is 22.49%, accounting for 19.7% of the global deaths [3]. Early, rational, and effective use of antibiotics is key to treat such patients [4–6]. Much evidence shows that optimizing antibiotic administration improves the clinical outcome of patients with sepsis [7–9]. A retrospective study [9] demonstrated that reasonable antibiotics can reduce the mortality of patients with sepsis [hazard ratio (HR) = 0.610, (95% confidence interval (CI): 0.433-0.858, *P*=0.005)]. The pharmacokinetics/pharmacodynamic(PK/PD) theory of antibiotics plays an important role in guiding the rational application of clinical antibiotics. However, due to a series of pathophysiological changes and life support measures, antibiotic PK often undergoes tremendous changes and fluctuates dynamically [10]. There are numerous factors caused by infection that change the antibiotic PK, such as capillary leakage, fluid resuscitation, the use of vasoactive drugs, tissue oedema, and changes in plasma protein binding rates [11].

Due to changes in PK, conventional dosages or methods of administration often failure to achieve a reasonable PK/PD target value, resulting in unsuccessful clinical treatment [8, 12]. A previous study [8] reported that in patients with severe sepsis or septic shock, EI of 1,000 mg of meropenem over 3 h administered Q8H is inadequate to provide activity (%fT > 4 µg/mL > 40%) against strains susceptible to increased exposure, requiring a bolus of 500 mg followed by EI of 1,500 mg Q8H. Vardakas et al. [12] pointed out that the mortality rate was significantly lower with long-term infusion of β-lactam for sepsis than with short-term infusion [risk ratio (RR) = 0.70, (95% confidence interval (CI): 0.56-0.87, *P*<0.005)]. Hence, long-term infusion or continuous infusion at a daily dose for 24 h with prolonged %fT> MIC might be helpful to reach the target PK/PD values.

Patients hospitalized in the ICU often require advanced life support, such as extracorporeal membrane oxygenation (ECMO), continuous renal replacement therapy (CRRT), etc. These supports often influence patients' PK, resulting in poor anti-infective efficacy and requiring antibiotic dose adjustment. Bougle et al. [13] showed that the majority of septic patients do not reach the PK target value during the adjuvant treatment of ECMO with imipenem and amikacin (60% and 66.7%, respectively). Similarly, due to increased antibiotic clearance during CRRT, especially for water-soluble antibiotics, it is critical to increase the dose to achieve the target values [14].

Therapeutic drug monitoring (TDM) can dynamically monitor the changes of drug concentration and optimize the antibiotic dosing regimens according to PK/PD target values [15]. Richter et al. [16] evaluated the compliance rate of PK target values under the TDM guidance for piperacillin/tazobactam during the treatment of sepsis and without TDM guidance; the rate of compliance with PK was significantly higher with TDM than without TDM. However, there are few antibiotics that can carry out TDM, which are limited to antibiotics with small therapeutic concentration range and obvious dose and adverse reactions, such as aminoglycosides, vancomycin, and antifungal drugs (e.g., echinocandins and flucytosine) [16, 17]. Therefore, adjusting antibiotics according to the pathophysiological characteristics of patients and PK characteristics of antibiotics mainly depends on ICU physicians’ experience in application of PK/PD. Therefore, ICU doctors' mastery of PK/PD knowledge is particularly important in the rational use of antibiotics.

Fleuren et al. [18] demonstrated that clinically relevant PK knowledge on antibiotic dosing among intensive care professionals is insufficient. This was addressed, as suboptimal dosing strategies were found to be associated with poorer outcomes. There are irrational use and abuse of antibiotics in China [19], and the drug resistance rate is remarkable [20]. More than 90% of macrolide-resistant *M. pneumoniae* are found in China. It is well-known that macrolide-resistant *M. pneumoniae* cause long-lasting fever, require long-term treatment, and lead to stronger side effects [20, 21], which might be related to the lack of clinicians’ PK/PD knowledge. Therefore, we carried out a cross-sectional study on ICU physicians’knowledge on PK/PD in China to assesse Chinese ICU physicians’knowledge about PK/PD, in order to emphasize the importance of antibiotic PK/PD knowledge in anti-infective treatment.

## Method

**Fig. 1:**
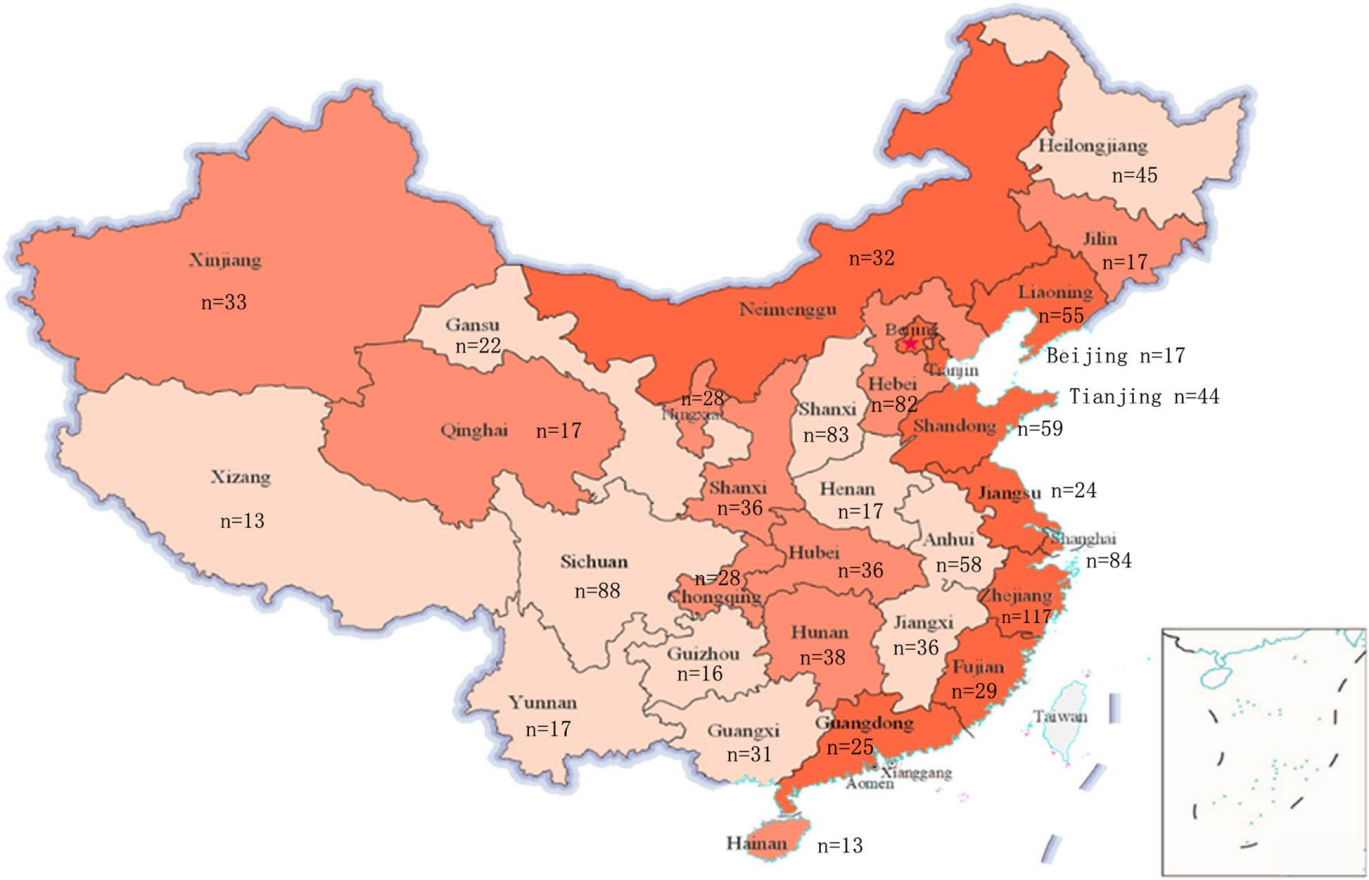
Distribution of participants in different provinces across mainland China

On December 24, 2019, we began a cross-sectional survey to assess ICU physicians’ PK/PD knowledge level. We designed a web-based questionnaire with www.wjx.cn, and all questions were jointly formulated by members of the Critical Care Medicine of Zhejiang Medical Association, China. The questionnaire link was shared via email and WeChat. Each recipient decided whether to answer the questions and whether to share the link with colleagues to get more responses to the questionnaire. The information collected included participants’ job title, years of work experience, and hospital grade. After participants completed the questionnaire, the results and other relevant information were available on the webpage. We summarized the information and analysed the questionnaires. Each participant was asked to complete the questionnaires according to their true knowledge level independently, without the help of mobile phones, computers, or other electronic devices. On March 24, 2020, we collected the questionnaire data and eliminated invalid questionnaires. Exclusion criteria were as follows: (1) the response duration was too short; there were 15 questions in total, and we excluded questionnaires with answering time less than 120 s as we believe that the respondents did not earnestly answer the questions according to their true knowledge; (2) the submitted answers for the 15 questions were all the same; and (3) when two or more questionnaires were answered from the same IP address, the last submission was retained. Finally, we totally collected 1,309 questionnaire results. After removing the invalid questionnaire, there were 1,240 valid questionnaires, and participants were distributed across 31 provinces in mainland China (Fig. 1).

### Designing questionnaire

We designed a total of 15 questions, each with a score of 10 points, and only one correct answer was acceptable, and all questions came from "Expert Consensus on Clinical Application of Antimicrobial Pharmacokinetics/Pharmacodynamics Theories", which is a Chinese expert consensus. The questions were related to the characteristics of antibiotics, PK/PD indices under different physiological and pathological conditions, and changes in PK/PD parameters under organ support and antibiotic adjustment, which are the most frequent problems that ICU physicians encounter clinically , and you can view the questionnaire content from the Supplementary File 1 "Questionnaire"

### Subjects

The subjects of this survey were ICU physicians who worked in a medical system in mainland China. We contacted 10,782 ICU physicians through 12th Congress of Chinese Society of Critical Care Medicine registry, including residents, attending physicians, vice chief physicians, and chief physicians. Resident physicians were defined as junior physicians who obtained qualification certificates for standardized resident training. Attending physicians, vice chief physicians, and chief physicians were defined as physicians who obtained relevant professional title evaluation examinations and met the requirements for professional title evaluation. The participants were from all grades of hospitals across the country. The hospitals grades were evaluated by the National Health Commission of the People’s Republic of China according to the medical service and management, medical quality and safety, technical level, and efficiency. The represented hospitals mainly included grade III-A, grade III-B, grade II-A, and grade II-B hospitals. Grade III-A hospitals refer to hospitals with more than 500 beds that provide medical and health services across regions, provinces, cities and the whole country, at the same time, more than 900 points were obtained according to the tertiary hospital grade scoring standard, and those with a comprehensive score of 750-899 are defined as grade III-B hospitals. Secondary hospitals are regional hospitals that provide medical and health services across several communities, with a total of 100 to 499 inpatient beds, and more than 900 points were obtained according to the secondary hospital grade scoring standard were defined as grade II-A hospitals, when those with a comprehensive score of 750-899 are defined as grade II-B hospitals. All the above information can be found on the official website of National Health Commission of the People’s Republic of China.

### Privacy and consent

We placed the informed consent form on the first page of the questionnaire, which included consent to publicly publish their answers, and strictly protected the private information of participants. Participants can only further answer the questionnaire content after clicking the "Agree" option, and choose "Disagree" to directly end the answer. The information we collected was limited to the hospital grade, years of working and the participant’s job title, and does not involve the participant’s name, age, work unit, home address, contact information. An institutional ethics board (Medical Ethics Committee of Zhejiang Hospital) approved the protocol (Approval Letter number: 2019-36K).

### The Angoff method

**Fig. 2:**
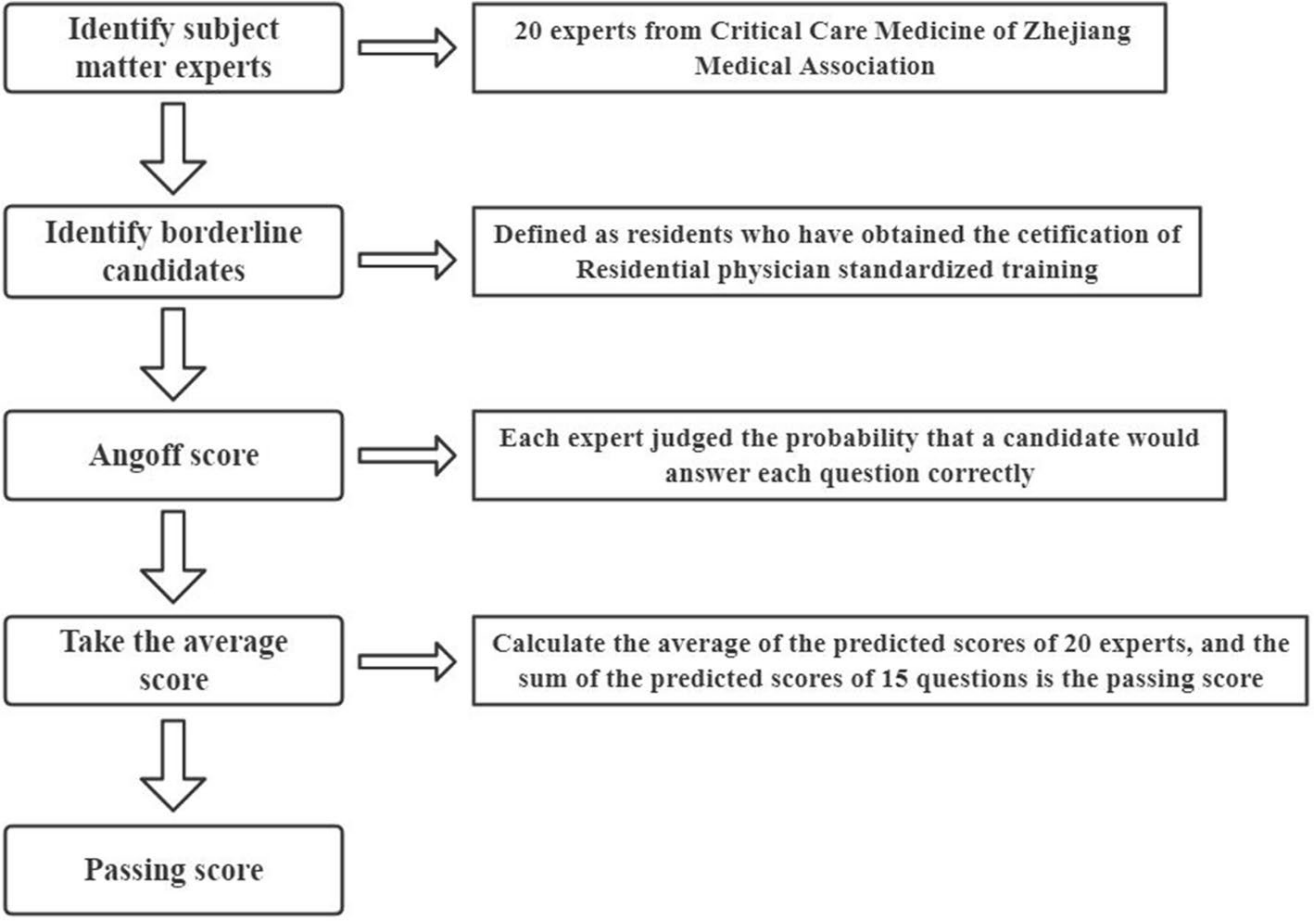
Correction of passing score by angooff method

To set the standard score, we used the Angoff method which uses a group of experts to judge the difficulty of each question on an exam to determine the cut-off score [22]. The Angoff method has been widely used in medical examination with high credibility [23–25]. We selected 20 members of Critical Care Medicine of Zhejiang Medical Association as the experts. First, we presented a lecture and reviewed PK/PD knowledge with the members of the committee, so they were prepared to judge the questionnaire. Each expert judged the probability that a candidate would answer each question correctly. We considered the average value to calculate the expected score for each question. The sum of the predicted scores for all questions represented the passing score (Fig. 2).

### Statistical analysis

The analysis was performed using Microsoft Excel (ver. 12624.20466). We imported all data into an Excel spreadsheet, analysed each questionnaire, performed a validity analysis, removed invalid data, and presented final results as images, tables, and text.

## Results

### Participants’ baseline characteristics

**Fig. 3:**
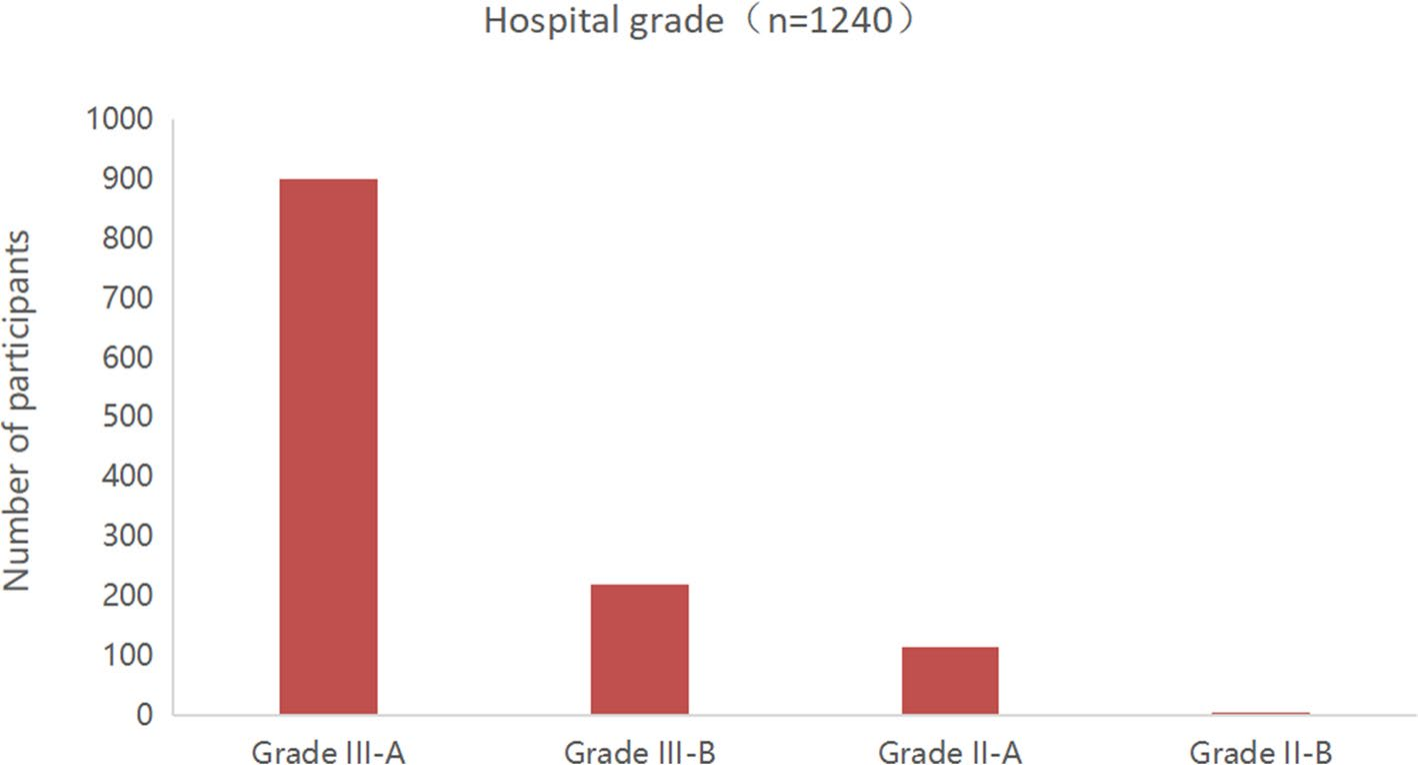
The grade distribution of the participants' hospitals

**Fig. 4:**
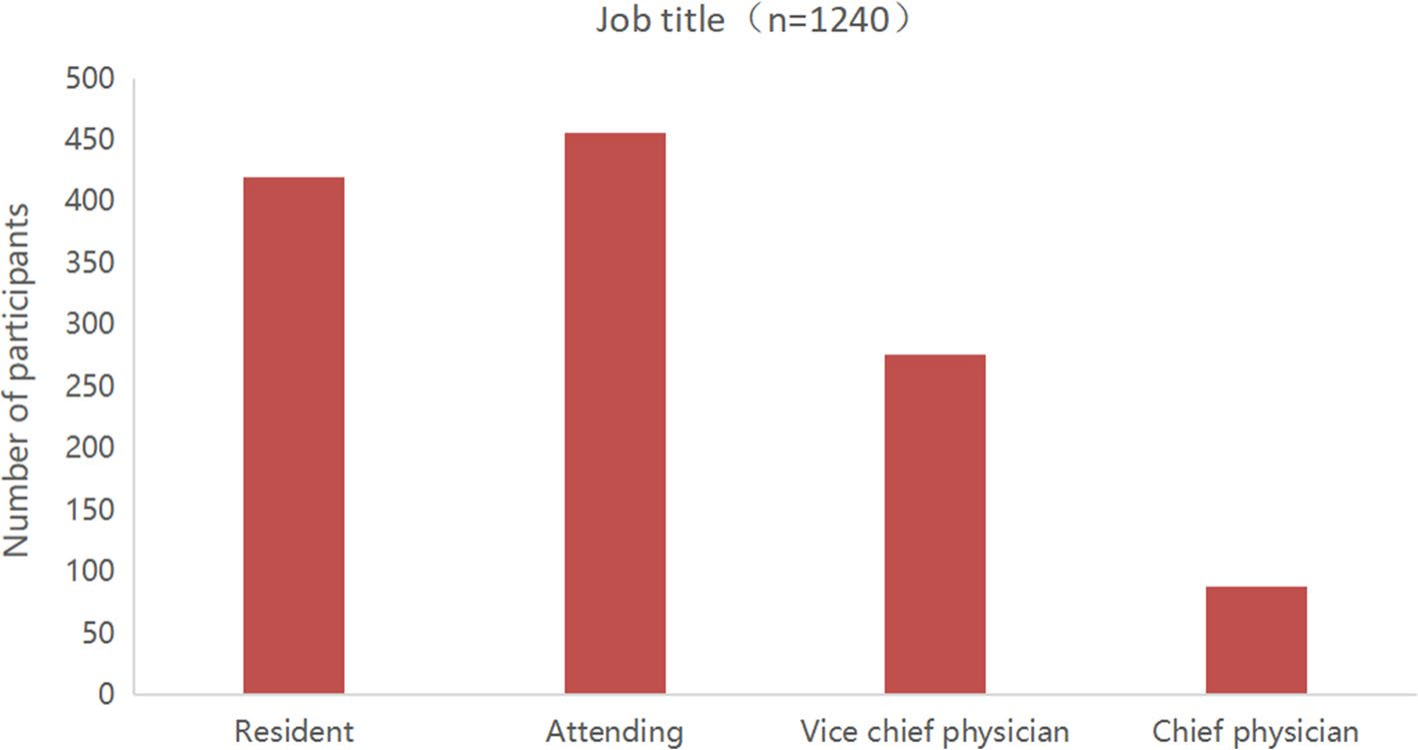
The distribution of the participants' job titles

**Fig. 5:**
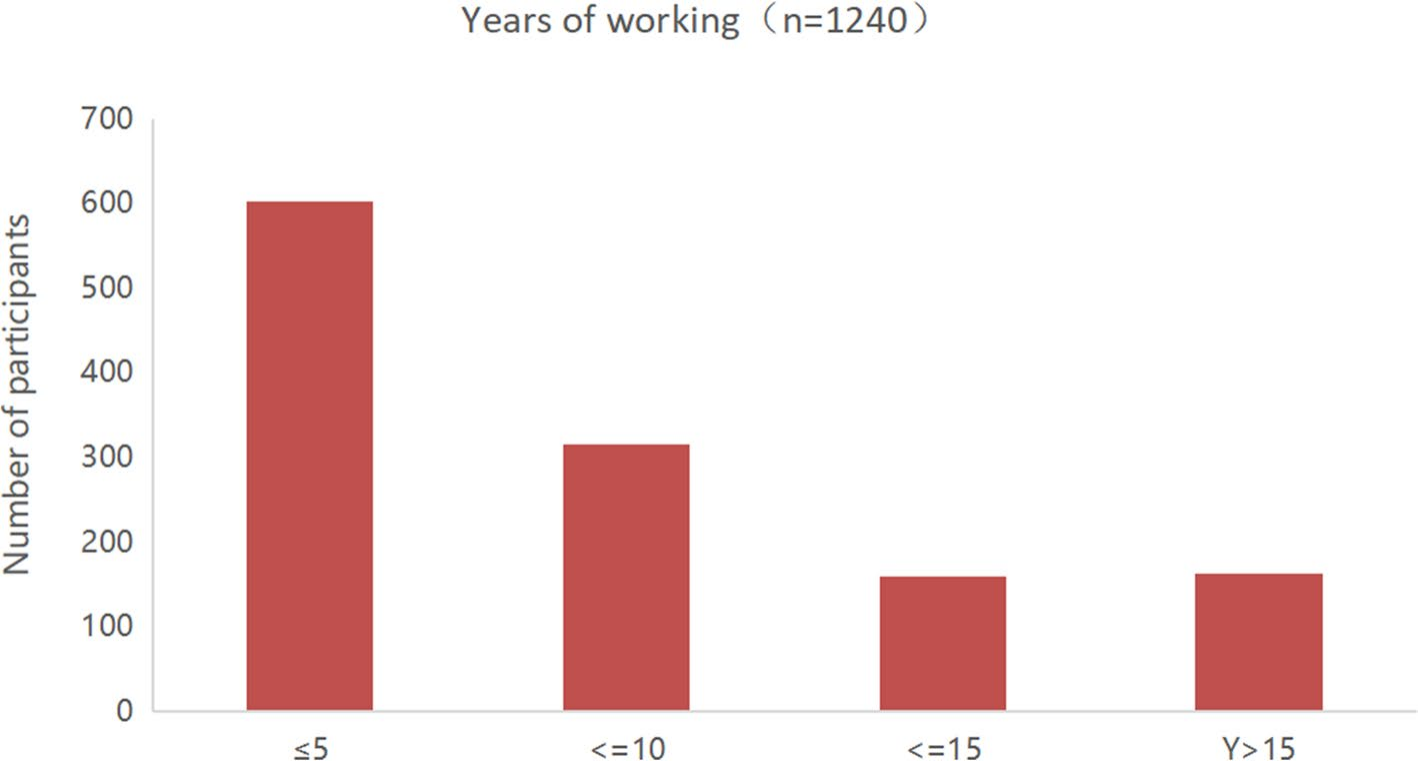
The distribution of participants' work experience

From December 24, 2019, to March 24, 2020, a total of 1,309 questionnaires were collected. After excluding invalid questionnaires, a total of 1,240 valid questionnaires remained. The participants were from 31 provinces of mainland China (Fig. 1). The subjects were mainly from grade III-A hospitals (*n*=900, 72.58%) (Fig. 3), and the majority of them were from Zhejiang province (*n*=117, 9.44%) (Fig. 1). Participants were mainly residents (*n*=420, 33.87%) and attending physicians (*n*=456, 36.77%) (Fig. 4), and the majority of them had work experience of no more than 10 years (*n*=919, 74.11%) (Fig. 5). Details of participants are shown in Table 1.

**Table 1:** Basic characteristics of participants

	Residents	Attending physicians	Vice chief physicians	Chief physicians	Total
Number of participants	420(33.9%)	456(36.7%)	276(22.3%)	88(7.1%)	1240
Years of working(Y)	2.2±1.6	6.8±3.1	13.9±3.9	15.3±5.3	7.4±5.8
Hospital grade					
Grade III-A	308(34.2%)	334(37.2%)	207(23.0)	51(5.6%)	900
Grade III-B	84(38.4%)	69(31.5%)	40(18.3%)	26(11.8%)	219
Grade II-A	26(22.4%)	50(43.1%)	29(25.0%)	11(9.5%)	116
Grade II-B	2(40.0%)	3(60.0%)	0(0.0%)	0(0.0%)	5

### Test results

**Fig. 6:**
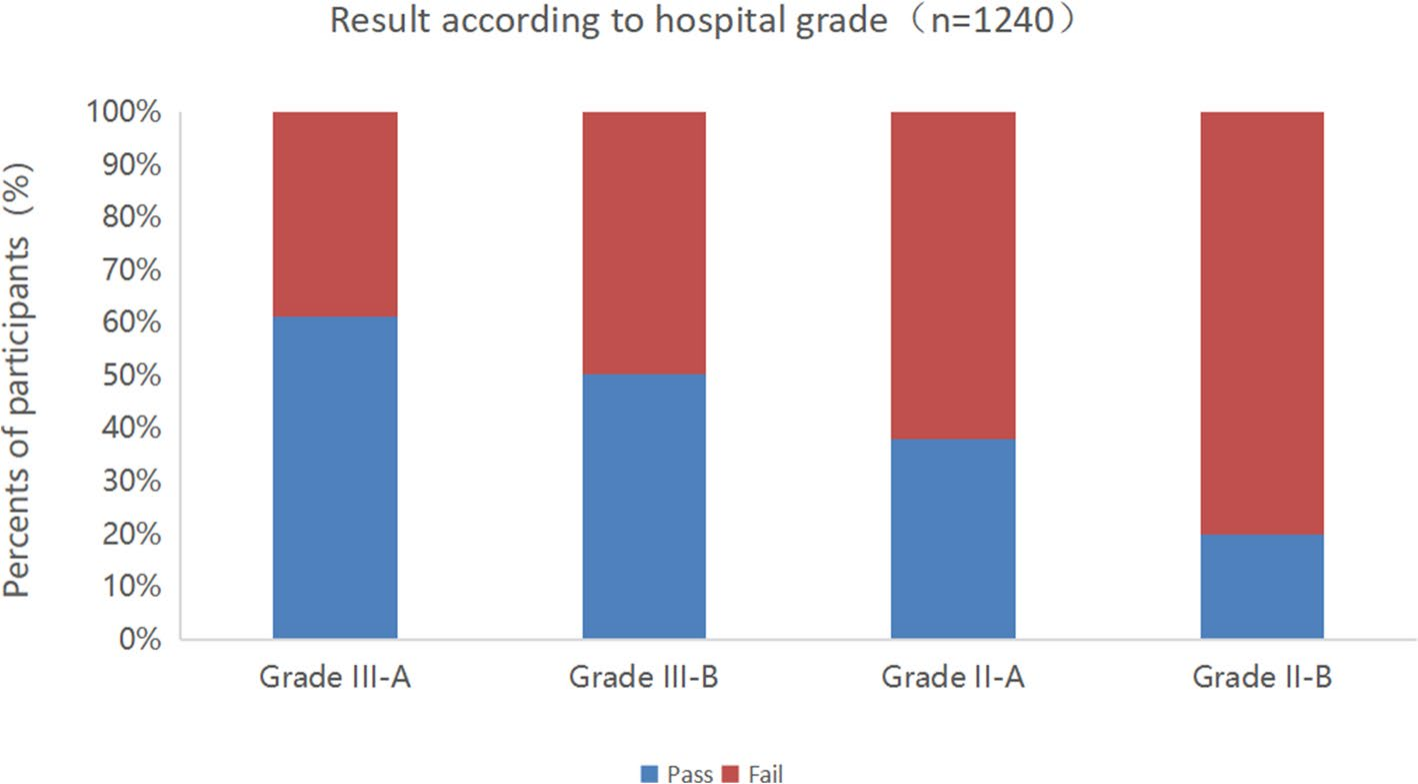
Test results according to hospital grade

**Fig. 7:**
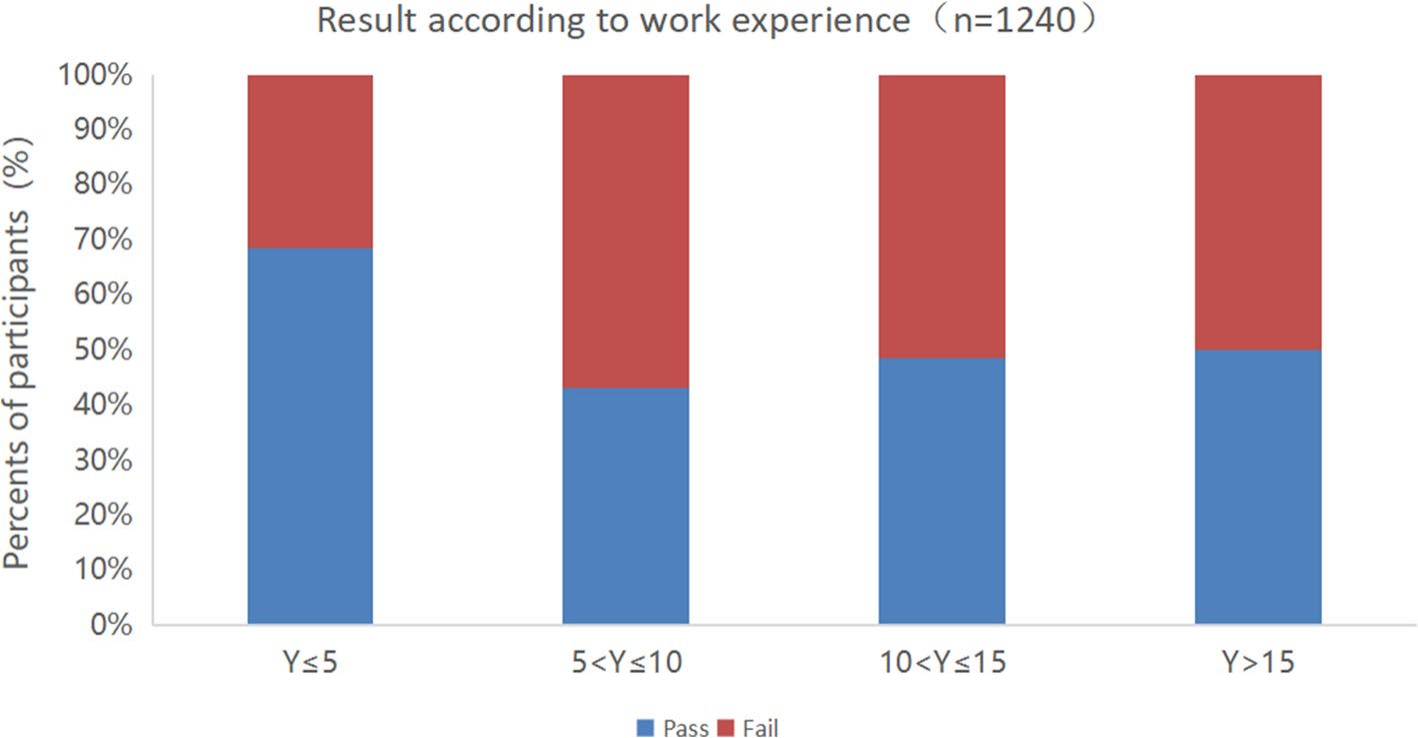
Test results according to work experience in the ICU

**Fig. 8:**
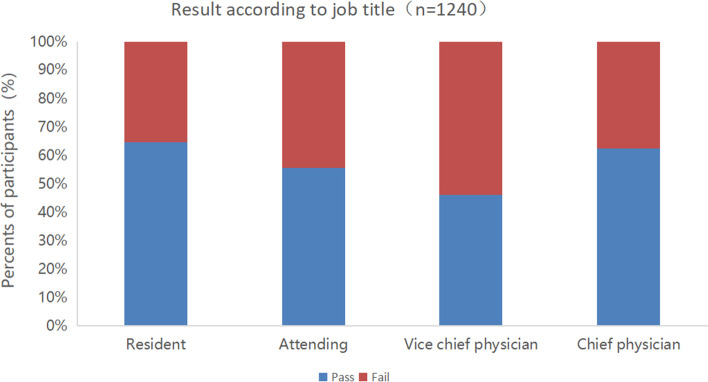
Test results according to job title

The passing score adjusted with the Angoff method was 90.8. Among 1,240 valid questionnaires, 706 (56.94%) participants passed the exam. The passing rate varied according to different characteristics of the physicians. From the perspective of hospital grade, higher hospital grade was associated with higher pass rate. The overall pass rate of the tertiary hospitals was 59.07%. Of the tertiary hospitals, the pass rate of the grade III-A hospitals was 61.22% and that of the grade III-B hospitals was 50.23%. The overall pass rate of the secondary hospitals was 37.19%. Of the secondary hospitals, the pass rate of the grade II-A hospitals was 37.93% (Fig. 6). From the perspective of work experience, ICU physicians with less than 5 years of work experience accounted for the highest pass rate, followed by physicians with work experience > 15 years and between 10 to 15 years (Fig. 7). From the perspective of physicians’ job-title, the pass rate of resident physician was the highest (64.52%), followed by chief physician, and attending physician, and vice chief physician for the lowest rate (Fig. 8).

### ICU physicians’ knowledge about PK

PK includes the processes of absorption, distribution, metabolism, and excretion of antibiotics [26]. We designed 10 questions about PK. Since the administration of antibiotics in patients with sepsis is mainly intravenous, our questions did not involve absorption-related knowledge of antibiotics. The antibiotic characteristic- (Q1-4), distribution- (Q6, Q7, Q11), and excretion-related knowledge (Q8, Q9) were covered in the questions. When the difficulty of the questions was low, the Angoff score was high (range, 0.6-0.8). Question 8 aimed to assess the knowledge related to metabolic pathways, and it had the highest correct rate (*n*=1134, 91.37%). Question 13 accounted for the lowest correct rate (*n* = 442, 35.65%), it required calculation of half-life which involves distribution- and excretion-related parameters. Therefore, the Angoff score and the pass rate of question 13 were low. The Angoff scores of the questions related to antibiotic characteristics were approximately 0.66, except for Question 1 which had a low correct rate (*n*=575, 46.37%). The correct rate of other topics was approximately 70-80%. The overall Angoff score of the antibiotic distribution-related questions was slightly lower; it was 48.47% for Question 7 and approximately 60% for the remaining questions, which suggested that ICU physicians’ knowledge was not ideal. Question 9 examined the route of antibiotic excretion, and less than half of the participants correctly answered this question (*n*=550, 44.35%), indicating a lack of ICU physicians’ knowledge of metabolic pathways (Table 2).

**Table 2:** Questions, standard answers, and Angoff score

Question	Answer	Correct answer	Angoff score	Pass rate
1. Which of the following antibiotics is liposoluble?	A. Ceftazidime B. Daptomycin C. Amikacin D. Moxifloxacin	D	0.645	46.37%
2. Which of the following antibiotics is water soluble?	A. Levofloxacin B. Imipenem C. Tegacyclin D. Lincomycin	B	0.675	72.58%
3. Which of the following antibiotics is a concentration-dependent antibiotic?	A. Meropenem B. Tegacyclin C. Nanofloxacin D. Ceftriaxone	C	0.670	79.60%
4. Which of the following antibiotics is time dependent?	A. Netilmicin B. Gatifloxacin C. Aztreonam D. Polymyxin	C	0.655	80.16%
5. Which of the following evaluation indicators is correct?	A. Ceftriaxone% T >mic B. Isopalmicin% T >mic C. MeropenemCmax/Mic D. TegacyclinCmax/Mic	A	0.585	69.44%
6. Which of the following is not a high plasma protein binding drug (PB> 70%)?	A. Tegacyclin B. Daptomycin C. Teicoplanin D. Fosfomycin	D	0.515	66.13%
7. Which of the following antibiotics does not need to be adjusted for hypoproteinaemia?	A. Cefoperazonesulbactam B. Doxycycline C. Teicoplanin D. Linezolid	D	0.590	48.47%
8. Which of the following antibiotics is mainly excreted through the kidney?	A. Minocycline B. Azithromycin C. Lincomycin D. Vancomycin	D	0.710	91.37%
9. Which of the following antibiotics is mainly excreted through the liver?	A. Imipenem/cilastatin B. Teicoplanin C. Clarithromycin D. Cefoperazone	D	0.600	44.35%
10. How should one adjust time-dependent antibiotics in critically ill patients?	A. Reduce dosage, increase frequency, and prolong infusion time B. Increase dosage, reduce frequency, and prolong infusion time C. Multiple daily doses, increase administration frequency, and prolong infusion time D. Single daily dose, increase dose, and extend infusion time	C	0.655	69.84%
11. Which of the following conditions will not lead to an increase in the apparent distribution volume (Vd) of antibiotics?	A. Hypoproteinemia B. Fluid resuscitation C. Severe infection D. Short bowel syndrome	D	0.595	78.47%
12. Which of the following statements is correct?	A. The main evaluation index of time-dependent antibiotic is% T >mic B. Auc0-24/MIC is the main evaluation index for concentration dependent antibiotic C. Cmax/MIC is the main evaluation index for time-dependent and PAE long antibiotics D. Time-dependent antibiotics with Cmax/MIC = 10-12	A	0.650	69.35%
13. What is the approximate half-life of an antibiotic with Vd of 200 L and CL of 10 L/h?	A. 12 h B. 14 h C. 16 h D. 18 h	B	0.485	35.65%
14. Which of the following antibiotics does not require dose adjustment during CRRT?	A. Water-soluble antibiotics B. Antibiotics with low plasma protein binding rate C. Antibiotics with high Vd D. Antibiotics with low molecular weight	C	0.550	75.56%
15. How should one adjust antibiotics with high Pb and low Vd during plasma exchange?	A. Extend infusion time B. Increase dosage C. Prolong infusion time and increase dosage D. Prolong interval of plasma exchange	D	0.500	38.31%

### ICU physicians’ knowledge about PD/PK and adjustment of antibiotic use

PD mainly reflects the effects of antibiotics on the body and clinical efficacy. We designed a total of 5 PK/PD questions, which mainly involved the adjustment of different types, periods, and dosages of antibiotics under organ support. The Angoff score was between 0.6-0.7, and the overall pass rate was approximately 70%. These questions mainly involved comprehensive indices related to antibiotics (Q5, Q12) as well as adjustment of dosage and administration methods under different pathological conditions (Q10, Q14-15). The question regarding adjustment of antibiotics under organ support was more difficult, especially that on the adjustment of plasma exchange antibiotics. The Angoff score was lower (0.500), and the pass rate was as low as 38.31%, which demonstrated that ICU physicians’ PD knowledge was not satisfactory (Table 2).

### Improving ICU physicians’ knowledge about PK/PD via professional training programmes

The Chinese Critical Care Certified Course (5C) is the most important training programme for medical staff in China. The training programme involves all aspects of the fields related to critical medicine and a programme on adjustment of antibiotic administration for critically ill patients according to PK/PD. And from 2009 to 2020, 5C training courses have been held 128 times nationwide, covering all provinces and autonomous regions. We compiled the number of ICU physicians who received this specific training programme in 31 provinces in mainland China from 2009 to 2019. In that period, 23,273 physicians participated in the programme and 18940 physicians passed the examination (Table 3). The majority of participants in the programme were from Jiangsu (*n*=1,808,9.55%) and Henan (*n*=1,599,8.44%) provinces, which had the highest average scores (125.8 and 126.5, respectively). However, for Beijing and Shanghai, the percentages of participants were low, and the average scores were low (79.4 and 90.9, respectively). Pearson correlation analysis showed that there was no correlation between the number of training participants and the passing rate of exams, and the percentage of registered physicians in 5C training and the passing rate of exams (*p*>0.05). The average test score of different provinces is positively correlated with the number of participants in the training (*r*=0.546, *p*=0.043), and the average test score is positively correlated with the percentage of 5C training physician registration (*r*=0.546, *p*=0.044). It shows that training can enhance ICU doctors' knowledge of antibiotic PK/PD.

**Table 3:** The details of ICU physicians who attended in 5C training programme

Provinces	Train-certified physicians, (%)	Average score		Pass rate (%)
Jiangsu	1808 (9.55)	125.8	100%	
Henan	1599 (8.44)	126.5	100%	
Guangdong	1428 (7.54)	102.4	44.0%	
Sichuan	1293 (6.83)	92.4	44.3%	
Zhejiang	1151(6.08)	71.6	32.4%	
Shanxi	820 (4.33)	106.1	47.2%	
Hebei	800 (4.22)	117.3	91.4%	
Shandong	669 (3.53)	85.4	37.2%	
Chongqing	653 (3.45)	70.0	28.5%	
Yunnan	617 (3.26)	110	76.4%	
Hubei	610 (3.22)	93.1	100%	
Hunan	598 (3.16)	86.5	100%	
Beijing	541 (2.86)	79.4	47.1%	
Shanghai	386 (2.04)	90.9	50.0%	
Others	5967 (31.50)	95.8	55.5%	

## Discussion

The present survey assessed the status of PK/PD knowledge among Chinese ICU physicians. The results showed that approximately half of the participants passed the exam, especially for questions on the adjustment of antibiotics for sepsis and the adjustment of antibiotics under organ support. Additionally, hospitals with different grades and physicians with different job titles exhibited different degrees of PK/PD knowledge. What surprises us is that the passing rate of resident physicians is the highest, which seems unreasonable, because generally the higher the working years, the higher the theoretical knowledge and professional level [27]. At the same time, the average score of chief physician and ICU physician who has worked for more than 15 years is the highest, but the average score is the highest, which indicates that there is "polarization" in the mastery level of PK/PD among physicians with the same job title, and the mastery situation is quite different. However, the total number of participants in this study is small, accounting for only about 1% of the total number of ICU practitioners in China. At the same time, the number of deputy chief physicians and chief physicians is small, which may have some bias. And combined with the results of 5C training, whether to accept continuing education training also has a certain impact on the test results. However, in general, the mastery of PK/PD knowledge of Chinese ICU doctors is unsatisfactory.

In addition to Chinese ICU physicians, those in other countries also lack acceptable of knowledge of PK/PD. A study conducted in Europe showed that European ICU professionals do not have a satisfactory level of PK knowledge [18]. Even though the theoretical knowledge is available in China, combined with the results of the current survey, we found that PK/PD knowledge has not been valued by clinicians. Given the high mortality rate of sepsis and increasing antibiotic resistance, methods to improve ICU physicians’ PK/PD knowledge need to be further studied.

Sepsis is a common reason for admission to ICUs, and early correct antibiotic use is an important measure to reduce mortality due to sepsis [9]. On the one hand, patients with sepsis may also have organ dysfunction, resulting in drug accumulation and adverse reactions. On the other hand, insufficient dose of antibiotics may lead to treatment failure and drug resistance [28]. Additionally, organ support may significantly influence PK/PD [29]. Therefore, for patients with sepsis, an optimal antibiotic administration strategy is essential to minimize the occurrence of adverse reactions and drug resistance while ensuring efficacy.

TDM of antimicrobials, even those with a wide therapeutic index, is becoming more common, whilst TDM for “traditional” drugs, such as aminoglycosides and glycopeptides, is continually being studied for further improvement. In the case of in vitro drug susceptibility testing with known pathogenic bacteria and minimum inhibitory concentration, TDM is carried out by monitoring changes in serum concentration combined with PK/PD target attainment [15]. An antibiotic-based adjustment programme remarkably improves treatment efficiency [30], reduces the occurrence of antibiotic adverse reactions, and reduces the mortality of septic patients [31, 32]. A recent TDM-based study [33] demonstrated that TDM should be routinely carried out for monitoring β-lactam antibiotics and aminoglycosides, while linezolid, teicoplanin, and vancomycin need to be monitored by TDM for critically ill patients. Using artificial intelligence-based methods such as Bayesian theory, PK/PD-relevant parameters can be calculated by combining blood drug concentration and patient's personal information, to achieve individualized antibiotic treatment [34–37]. Studies have shown that Bayesian theory combined with TDM can accurately monitor the dosage of antibiotics [38, 39].

At the same time, multidisciplinary cooperation can also optimize the antibiotic dosing regimen; strengthening the cooperation of clinicians, nurses, pharmacists, infection departments, microbiology rooms, and other departments can greatly improve the efficiency of drug delivery [40]. Richter et al. [41] pointed out that implementation of TDM in sepsis patients under the premise of multidisciplinary cooperation can improve the stability of plasma antibiotic levels, shorten the treatment time, and improve treatment effectiveness. After multidisciplinary cooperation, clinicians’ decision-making efficiency was elevated by 90% [42]. Another study on the multidisciplinary cooperative administration of aminoglycosides showed that ensuring the effective concentration of aminoglycosides also reduces the toxicity and side effects of drugs [43].

At present, the best method for ICU physicians to optimize antibiotic delivery is to enhance their PK/PD-based knowledge. Our survey suggested that ICU physicians’ PK/PD knowledge was not satisfactory in China. The distribution of antibiotics mainly depends on the volume of distribution (Vd) and the binding rate of plasma protein (PB). Vd is a theoretical volume affected by infection, protein level, and renal function [44]; comprehension of Vd is challenging for ICU physicians. The plasma protein binding rate is closely associated with the protein level in patients [45]. The efficacy of high binding rate is often affected, but it does not attract ICU physicians’ attention. The half-life of antibiotics is affected by the apparent volume of distribution and drug clearance [5]. Due to the complicated condition, it is difficult to calculate the half-life of drugs in patients with sepsis in clinical practice; thus, the rate of correct usage of antibiotics is low. Additionally, there are several other factors that affect PK in septic patients, and it is critical to understand the characteristics of antibiotics and comprehensively analyse the pathophysiological changes in septic patients.

Training is one of the most important approaches to enhance ICU physicians’ PK/PD knowledge. Our previous research revealed that the success of COVID-19 treatment is related to whether medical staff received professional training [46]. In the present survey, the advantages of professional training programmes for ICU physicians were discussed. The greater the number of participants in training programme, the greater the knowledge of PK/PD. However, there are remarkable gaps between the 5C training programme and knowledge-based PK/PD in different regions of mainland China. These results indicate that further attention should be paid to enhance ICU physicians’ knowledge about PK/PD.

This study has some limitations. First, the sample size was small, and only 1% of the total Chinese ICU staff were included. Second, ICU physicians who participated in the survey were from tertiary hospitals, and the number of physicians belonging to secondary hospitals was relatively limited. Third, the participants were mainly from the developed regions of eastern China, and there were fewer participants from western regions. Fourth, we contacted 10,782 ICU physicians through 12th Congress of Chinese Society of Critical Care Medicine registry, the sampling is not representative, there is a certain randomness in the sampling, and the sampling error is large, which can not represent the actual situation of the whole country.

## Conclusion

ICU physicians’ knowledge of PK/PD is not satisfactory. Improving ICU physicians’ knowledge about PK/PD is critical. Additionally, further implementation of TDM for testing drug concentrations and developing real-time medication software in conjunction with other departments is encouraged.

## Supplementary Information

**Additional file 1.**

## Data Availability

The completed questionnaires were collected and safely stored in the principal investigator’s office. Data were saved in an Excel spread sheet. The data sets used and analysed are available from the corresponding author upon reasonable request.

## Abbreviations

ICU: Intensive care unit
PK: Pharmacokinetics
PD: Pharmacodynamics
MIC: Minimum inhibitory concentration
Cmax: Peak concentration
AUC0-24: Area under the cure
%fT>MIC: %FT>Minimum inhibitory concentration
ECMO: Extracorporeal membrane oxygenation
CRRT: Continuous renal replacement therapy
TDM: Therapeutic drug monitoring
IP: Internet Protocol Address
PB: Binding rate of plasma protein
Vd: Apparent volume of distribution
CL: Clearance rate
5C: Chinese Critical Care Certified Course
ESICM: Infection Section of European Society of Intensive Care Medicine

## References

[1] Fleischmann C, Scherag A, Adhikari NK (2016). Assessment of Global Incidence and Mortality of Hospital-treated Sepsis. Current Estimates and Limitations. Am J Respir Crit Care Med.

[2] Rhee C, Klompas M (2020). Sepsis trends: increasing incidence and decreasing mortality, or changing denominator?. J Thorac Dis.

[3] Rudd KE, Johnson SC, Agesa KM (2020). Global, regional, and national sepsis incidence and mortality, 1990–2017: analysis for the Global Burden of Disease Study. Lancet.

[4] Schinkel M, NannanPanday RS, Wiersinga WJ (2020). Timeliness of antibiotics for patients with sepsis and septic shock. J Thorac Dis.

[5] Roberts JA, Abdul-Aziz MH, Lipman J (2014). Individualised antibiotic dosing for patients who are critically ill: challenges and potential solutions. Lancet Infect Dis.

[6] Lindberg O, De Geer L, Chew MS (2020). Nonadherence to antibiotic guidelines in patients admitted to ICU with sepsis is associated with increased mortality: A registry-based, retrospective cohort study. Eur J Anaesthesiol.

[7] Ahmed N, Jen SP, Altshuler D, et al. Evaluation of Meropenem Extended Versus Intermittent Infusion Dosing Protocol in Critically Ill Patients. J Intensive Care Med. 2020;35(8):763-771. 10.1177/0885066618784264 29954243

[8] Kothekar AT, Divatia JV, Myatra SN (2020). Clinical pharmacokinetics of 3-h extended infusion of meropenem in adult patients with severe sepsis and septic shock: implications for empirical therapy against Gram-negative bacteria. Ann Intensive Care.

[9] Al-Sunaidar KA, Aziz NPA, Hassan YP (2020). Appropriateness of empirical antibiotics: risk factors of adult patients with sepsis in the ICU. Int J Clin Pharm.

[10] Roberts JA, Lipman J (2009). Pharmacokinetic issues for antibiotics in the critically ill patient. Crit Care Med.

[11] Udy AA, Roberts JA, De Waele JJ (2012). What's behind the failure of emerging antibiotics in the critically ill? Understanding the impact of altered pharmacokinetics and augmented renal clearance. Int J Antimicrob Agents.

[12] Vardakas KZ, Voulgaris GL, Maliaros A (2018). Prolonged versus short-term intravenous infusion of antipseudomonal β-lactams for patients with sepsis: a systematic review and meta-analysis of randomised trials. Lancet Infect Dis.

[13] Bougle A, Dujardin O, Lepere V (2019). PHARMECMO: Therapeutic drug monitoring and adequacy of current dosing regimens of antibiotics in patients on Extracorporeal Life Support. Anaesth Crit Care Pain Med.

[14] Jang SM, Infante S, Abdi PA (2020). Drug Dosing Considerations in Critically Ill Patients Receiving Continuous Renal Replacement Therapy. Pharmacy (Basel).

[15] Sima M, Bakhouche H, Hartinger J (2019). Therapeutic drug monitoring of antibiotic agents: evaluation of predictive performance. Eur J Hosp Pharm.

[16] Richter DC, Frey O, Rohr A (2019). Therapeutic drug monitoring-guided continuous infusion of piperacillin/tazobactam significantly improves pharmacokinetic target attainment in critically ill patients: a retrospective analysis of four years of clinical experience. Infection.

[17] Muller AE, Huttner B, Huttner A (2018). Therapeutic Drug Monitoring of Beta-Lactams and Other Antibiotics in the Intensive Care Unit: Which Agents, Which Patients and Which Infections?. Drugs.

[18] Fleuren LM, Roggeveen LF, Guo T (2019). Clinically relevant pharmacokinetic knowledge on antibiotic dosing among intensive care professionals is insufficient: a cross-sectional study. Crit Care.

[19] Yin X, Song F, Gong Y (2013). A systematic review of antibiotic utilization in China. J Antimicrob Chemother.

[20] Qiao M, Ying GG, Singer AC (2018). Review of antibiotic resistance in China and its environment. Environ Int.

[21] Hu F, Zhu D, Wang F (2018). Current Status and Trends of Antibacterial Resistance in China. Clin Infect Dis.

[22] Dwyer T, Wright S, Kulasegaram KM (2016). How to set the bar in competency-based medical education: standard setting after an Objective Structured Clinical Examination (OSCE). BMC Med Educ.

[23] Verheggen MM, Muijtjens AM, Van Os J (2008). Is an Angoff standard an indication of minimal competence of examinees or of judges?. Adv Health Sci Educ Theory Pract.

[24] Mubuuke AG, Mwesigwa C, Kiguli S (2017). Implementing the Angoff method of standard setting using postgraduate students: Practical and affordable in resource-limited settings. Afr J Health Prof Educ.

[25] Karcher C (2019). The Angoff method in the written exam of the College of Intensive Care Medicine of Australia and New Zealand: setting a new standard. Crit Care Resusc.

[26] Sy SK, Zhuang L, Derendorf H (2016). Pharmacokinetics and pharmacodynamics in antibiotic dose optimization. Expert Opin Drug Metab Toxicol.

[27] Gong YJ, Hong T, Jiang J (2012). Influence of education and working background on physicians' knowledge of secondary prevention guidelines for coronary heart disease: results from a survey in China. J Zhejiang Univ Sci B.

[28] Fujii M, Karumai T, Yamamoto R (2020). Pharmacokinetic and pharmacodynamic considerations in antimicrobial therapy for sepsis. Expert Opin Drug Metab Toxicol..

[29] Annoni F, Grimaldi D, Taccone FS (2020). Individualized antibiotic therapy in the treatment of severe infections. Expert Rev Anti Infect Ther.

[30] Brinkmann A, Rohr AC, Koberer A (2018). Therapeutic drug monitoring and individual dosing of antibiotics during sepsis : Modern or just "trendy"?. Med Klin Intensivmed Notfmed.

[31] Hagel S, Fiedler S, Hohn A (2019). Therapeutic drug monitoring-based dose optimisation of piperacillin/tazobactam to improve outcome in patients with sepsis (TARGET): a prospective, multi-centre, randomised controlled trial. Trials.

[32] Tangden T, Ramos Martin V, Felton TW (2017). The role of infection models and PK/PD modelling for optimising care of critically ill patients with severe infections. Intensive Care Med.

[33] Abdul-Aziz MH, Alffenaar JC, Bassetti M (2020). Antimicrobial therapeutic drug monitoring in critically ill adult patients: a Position Paper. Intensive Care Med.

[34] Delattre IK, Musuamba FT, Nyberg J (2010). Population pharmacokinetic modeling and optimal sampling strategy for Bayesian estimation of amikacin exposure in critically ill septic patients. Ther Drug Monit.

[35] Avent ML, Rogers BA (2019). Optimising antimicrobial therapy through the use of Bayesian dosing programs. Int J Clin Pharm.

[36] Broeker A, Nardecchia M, Klinker KP (2019). Towards precision dosing of vancomycin: a systematic evaluation of pharmacometric models for Bayesian forecasting. Clin Microbiol Infect.

[37] Donagher J, Martin JH, Barras MA (2017). Individualised medicine: why we need Bayesian dosing[J]. Intern Med J.

[38] Roggeveen LF, Fleuren LM, Guo T (2019). Right Dose Right Now: bedside data-driven personalized antibiotic dosing in severe sepsis and septic shock - rationale and design of a multicenter randomized controlled superiority trial. Trials.

[39] Lipman J (2017). Right Dose, Right Now: Customized Drug Dosing in the Critically Ill. Crit Care Med.

[40] Lynch TJ, Possidente CJ, Cioffi WG, Hebert JC (1992). Multidisciplinary protocol for determining aminoglycoside dosage. Am J Hosp Pharm.

[41] Richter DC, Heininger A, Brenner T (2019). Bacterial sepsis : Diagnostics and calculated antibiotic therapy. Anaesthesist.

[42] Yodoshi M, Iwasaki N, Satoh K (2013). TDM management system for contribution to proper use of anti-mRSA drugs–establishment of cooperation support system between pharmacy and clinical laboratory in hospital. Rinsho Byori.

[43] Lynch TJ, Possidente CJ, Cioffi WG, Hebert JC (1992). Multidisciplinary protocol for determining aminoglycoside dosage. Am J Hosp Pharm.

[44] Pea F (2018). Intracellular Pharmacokinetics of Antibacterials and Their Clinical Implications. Clin Pharmacokinet.

[45] Ulldemolins M, Roberts JA, Rello J (2011). The effects of hypoalbuminaemia on optimizing antibacterial dosing in critically ill patients[J]. Clin Pharmacokinet.

[46] Li L, Xv Q, Yan J (2020). COVID-19: the need for continuous medical education and training. Lancet Respir Med.

