# Supplementary material for: Chinese ICU physicians’ knowledge of antibiotic pharmacokinetics/pharmacodynamics (PK/PD): a cross-sectional survey
Source: BMC Med Educ. 2022 Mar 14;22:173. doi: 10.1186/s12909-022-03234-9 (PMC8920424; doi:10.1186/s12909-022-03234-9)
Supplement: Supplementary file 1 — Additional file 1. [file 12909_2022_3234_MOESM1_ESM.docx]

Zhejiang Provincial Critical Care Research Group (ZJCCR Group) Invitation letter of "Chinese ICU physicians’knowledge of antibiotic pharmacokinetics/pharmacodynamics (PK/PD): A cross-sectional survey"


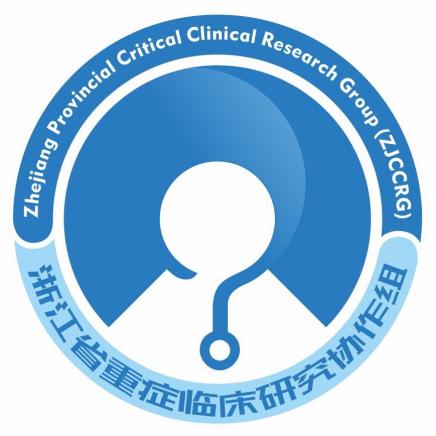


Dear experts

The situation of bacterial drug resistance is becoming more and more serious, We need to optimize the use of antibiotics according to the pharmacokinetics(PK) and pharmacodynamics(PD). The PK/PD of antibiotics in severe patients often changes significantly, and it is more necessary to optimize the use of antibiotics based on the PK/PD characteristics. In order to deeply understand the knowledge of domestic ICU doctors on PK/PD, Zhejiang Provincial Critical Care Research Group (ZJCCR Group) sincerely invites you to join our research and complete the following questionnaire. And the information we collected include the hospital grade, years of working and job title, we will strictly protecte the private information of participants. When you receive this link, in the first page, you should click the "Agree" option to start your answer, and choose "Disagree" to directly end the answer.

Thank you!

Yours sincerely

Decide whether to further participate in the questionnaire according to your wishes.

- Agree
- Disagree

**Essential information**

Your job title?

1. Residents
2. Attending physicians
3. Vice chief physicians
4. Chief physicians

Years of work experience

1. Y≤5
2. 5＜Y≤10
3. 10＜Y≤15
4. Y＞15

The hospital grade of your working hospital

1. Grade III-A
2. Grade III-B
3. Grade II-A
4. Grade II-B

Which province are you from_______________

**Questionnaire**

1. Which of the following antibiotics is liposoluble?

A. Ceftazidime

B. Daptomycin

C. Amikacin

D. Moxifloxacin

1. Which of the following antibiotics is water soluble?

A. Levofloxacin

B. Imipenem

C. Tegacyclin

D. Lincomycin

1. Which of the following antibiotics is a concentration-dependent antibiotic?

A. Meropenem

B. Tegacyclin

C. Nanofloxacin

D. Ceftriaxone

1. Which of the following antibiotics is time dependent?

A. Netilmicin

B. Gatifloxacin

C. Aztreonam

D. Polymyxin

1. Which of the following evaluation indicators is correct?

A. Ceftriaxone% T >mic

B. Isopalmicin% T >mic

C. MeropenemCmax/Mic

D. TegacyclinCmax/Mic

1. Which of the following is not a high plasma protein binding drug (PB> 70%)?

A. Tegacyclin

B. Daptomycin

C. Teicoplanin

D. Fosfomycin

1. Which of the following antibiotics does not need to be adjusted for hypoproteinaemia?

A. Cefoperazonesulbactam

B. Doxycycline

C. Teicoplanin

D. Linezolid

1. Which of the following antibiotics is mainly excreted through the kidney?

A. Minocycline

B. Azithromycin

C. Lincomycin

D. Vancomycin

1. Which of the following antibiotics is mainly excreted through the liver?

A. Imipenem/cilastatin

B. Teicoplanin

C. Clarithromycin

D. Cefoperazone

1. How should one adjust time-dependent antibiotics in critically ill patients?

A. Reduce dosage, increase frequency, and prolong infusion time

B. Increase dosage, reduce frequency, and prolong infusion time

C. Multiple daily doses, increase administration frequency, and prolong infusion time

D. Single daily dose, increase dose, and extend infusion time

1. Which of the following conditions will not lead to an increase in the apparent distribution volume (Vd) of antibiotics?

A. Hypoproteinemia

B. Fluid resuscitation

C. Severe infection

D. Short bowel syndrome

1. Which of the following statements is correct?

A. The main evaluation index of time-dependent antibiotic is% T >mic

B. Auc0-24/MIC is the main evaluation index for concentration dependent antibiotic

C. Cmax/MIC is the main evaluation index for time-dependent and PAE long antibiotics

D. Time-dependent antibiotics with Cmax/MIC = 10-12

1. What is the approximate half-life of an antibiotic with Vd of 200 L and CL of 10 L/h?
2. 12 h
3. 14 h
4. 16 h
5. 18 h
6. Which of the following antibiotics does not require dose adjustment during CRRT?

A. Water-soluble antibiotics

B. Antibiotics with low plasma protein binding rate

C. Antibiotics with high Vd

D. Antibiotics with low molecular weight

1. How should one adjust antibiotics with high Pb and low Vd during plasma exchange?

A. Extend infusion time

B. Increase dosage

C. Prolong infusion time and increase dosage

D. Prolong interval of plasma exchange
